# Left ventricular free wall rupture caused by myocardial ischemia without treatable atherosclerotic coronary disease: a case series

**DOI:** 10.1186/s13019-024-02690-2

**Published:** 2024-04-12

**Authors:** Terézia B. Andrási, Nunijiati Abudureheman, Alannah C. Glück, Kai Dielmann, Gerhard Dinges

**Affiliations:** 1 Department of Cardiac and Cardiovascular Surgery, Philipps University of Marburg, Baldingerstrasse 1, 35041 Marburg, Germany; 2 School of Medicine, Philipps University of Marburg, Marburg, Germany; 3 Department of Anesthesiology and Intensive Care Medicine, Philipps University of Marburg, Marburg, Germany

**Keywords:** Myocardial infarction, Left ventricular rupture, Resuscitation, Surgery

## Abstract

**Background:**

The clinical presentation of left ventricular free wall rupture (LVFWR) varies ranging from uneventful condition to congestive heart failure.

**Case summary:**

Here we report two cases of LVFWR with different clinical presentation and notable outcome. A 53-year-old male presenting emergently with signs of myocardial infarction received immediate coronary angiography and thoracic CT-scan showing occlusion of the first marginal coronary branch without possibility of revascularization and minimal pericardial extravasation. Under ICU surveillance, LVFWR occurred 24 h later and was treated by pericardiocentesis and ECMO support followed by immediate uncomplicated surgical repair. Postoperative therapy-refractory vasoplegia and electromechanical dissociation caused fulminant deterioration and the early death of the patient. The second case is a 76-year old male brought to the emergency room after sudden syncope, clinical sings of pericardial tamponade and suspicion of a type A acute aortic dissection. Immediate CT-angiography excluded aortic dissection and revealed massive pericardial effusion and a hypoperfused myocardial area on the territory of the first marginal branch. Immediate sternotomy under mechanical resuscitation enabled removal of the massive intrapericardial clot and revealed LVFWR. After an uncomplicated surgical repair, an uneventful postoperative course, the patient was discharged with sinus rhythm and good biventricular function. One year after the operation, he is living at home, symptom free.

**Discussion:**

Whereas the younger patient, who was clinically stable at hospital admission received delayed surgery and did not survive treatment, the older patient, clinically unstable at presentation, went into immediate surgery and had a flawless postoperative course. Thus, early surgical repair of LVFWR leads to best outcome and treating LVFWR as a high emergency regardless of the symptoms improve survival.

## Introduction

Primary percutaneous coronary interventions considerably reduced the rates of left ventricular free wall rupture (LVFWR) [1]. However, mortality after LVFWR continues to range between 8% and 35%, that is 8–10 times higher than for other types of myocardial discontinuity such as rupture of papillary muscle or of interventricular septum [1, 2].

Choosing the optimal therapy [3, 4] is challenged by the clinical presentation of LVFWR that varies ranging from uneventful condition to congestive heart failure. A discontinuity of the ventricular wall or a distinctive bi-directional flow between the extracardiac echo-free space and the left ventricle are rarely seen [5, 6]. Although cardiac-MRI [7] may allow a more precise differentiation between free rupture and a pseudoaneuryms, its effect on outcome is somewhat uncertain.

Thus, timing and invasiveness of the most successful treatment for LVFWR remains a subject of debate.

## Case presentations

### Patient I

A 53 year-old male patient presented at the emergency department with unstable angina pectoris since 24 h. He experienced similar symptoms a week before presentation when he had a cough and signs of pneumonia successfully treated with 600 mg Ibuprofen 3-times daily. He is smoker and his father died at the age of 53 due to acute myocardial infarction. Upon admission, the patient is febrile (38.5 °C) with respiratory frequency of 12/min, peripheral O_2_ saturation 96% under room air, sinus rhythm (77 beats/min) and blood pressure of 128/79 mmHg. The pH is 7.341, arterial lactate 0.4 mmol/L and venous lactate 1.6 mmol/L. High-sensitivity cardiac troponin I is 3600 ng/L and CK-MB is 40 U/L.

**Fig. 1:**
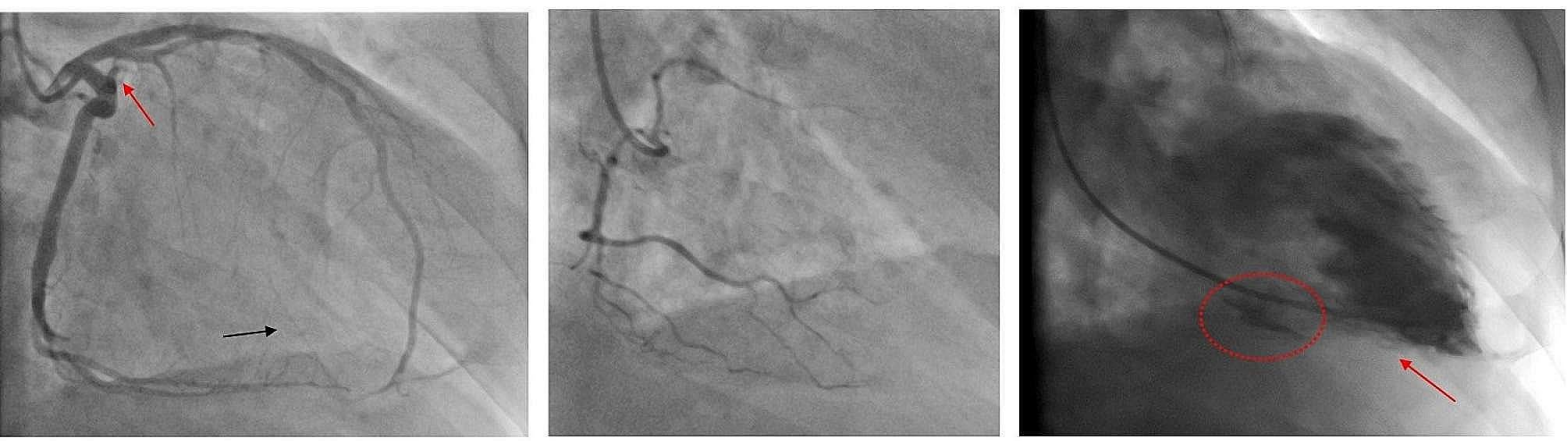
Preoperative screening for coronary disease in patient I Preoperative coronary angiography of patient I reveals occlusion of the first marginal branch (red arrow) with retrograde perfusion from the posterior interventricular branch (black arrow) of the left dominant coronary system (Fig. 1A), intact small right coronary system (Fig. 1B) and the suspicion of left ventricular pseudoaneuryms (red circle) without pericardial effusion of contrast substance (red arrow) in ventriculography (Fig. 1C)

**Fig. 2:**
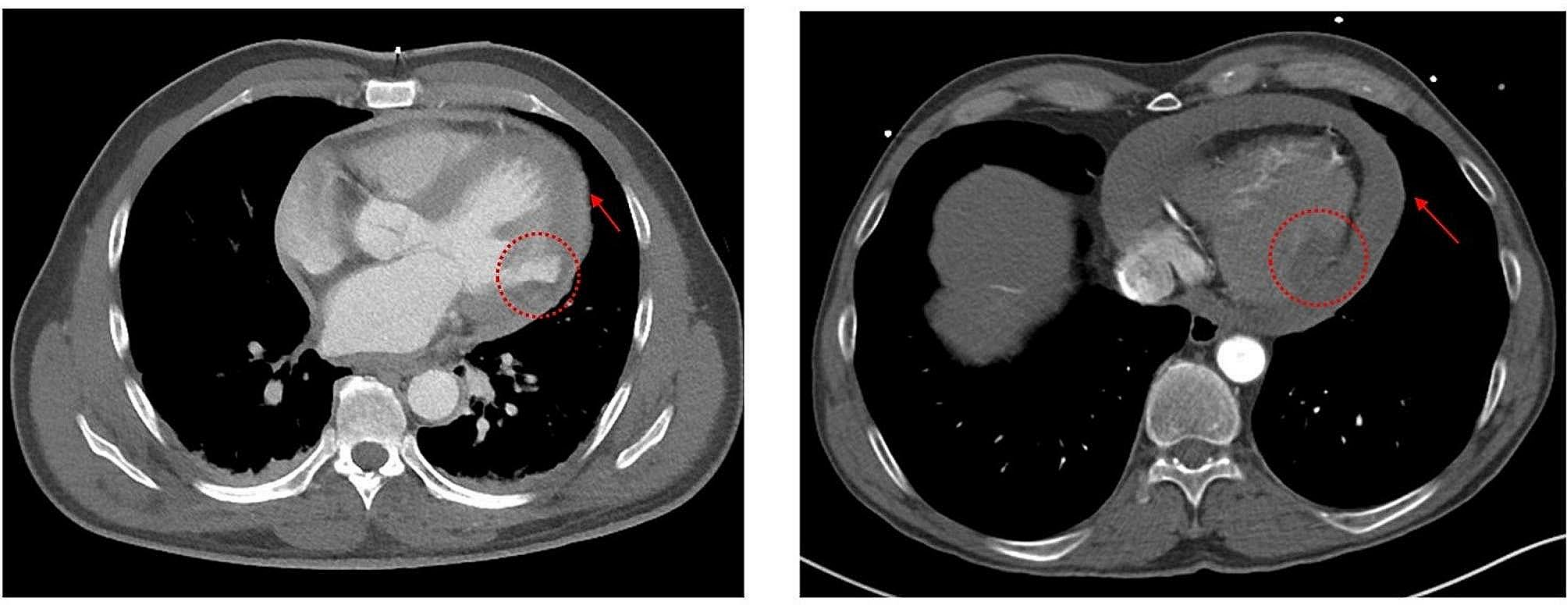
Preoperative thoracic CT-Angiogram Fig. 2A shows minimal pericardial effusion (red arrow) with suspicion of LVFWR of the first marginal branch (red circle) in patient I Fig. 2B shows relevant pericardial effusion (red arrow) and a hypocontrasted myocardial area of the first marginal branch (red circle) in patient II

Coronary angiography revealed an abrupt occlusion of the first marginal branch of the circumflex artery (Fig. 1A) without collateralization and an otherwise intact left dominant coronary artery system (Fig. 1B). Ventriculography raised the suspicion of a small left ventricular pseudoaneurysm (Fig. 1C). Transthoracic echocardiography revealed normal left ventricular function and volumes, left lateral wall dyskinesia and a small pericardial effusion. The CT angiography (Fig. 2A) confirmed the suspicion of small anterolateral left ventricular pseudo-aneurysm (red circle) without sign of free rupture and a minor pericardial effusion (red arrow).

The patient remained hemodynamically stable and clinically asymptomatic during and after the examinations and was transferred to the ICU for continuous surveillance. High-sensitivity Troponin I decreases from 3600 ng/L to 2300ng/L and CK-MB from 40 U/L to 26 U/L. Pro-BNP increases from 693 pg/mL to 1156 pg/mL (Table 1). The patient remained awake, asymptomatic, catecholamine-free and pulmonary stable with 2 L nasal oxygen. Cardiac MRI was planned for the next morning. Twenty hours after hospital admission, hemodynamic instability occurred followed by instant circulatory collapse. Cardiopulmonary resuscitation was initiated with intubation and mechanical ventilation. Ventricular fibrillation could not be converted to sinus rhythm. Echocardiography revealed relevant pericardial effusion. Pericardial drainage was performed and more than 1 L fresh arterial blood removed. Persistent electromechanical dissociation was assessed. Veno-arterial ECMO was placed through percutaneous femoral approach under massive volume resuscitation (2500 mL crystalloid). High-dose cathecolamine support (120 µg/min Noradrenalin) was required to maintain circulation. The patient was immediately transferred to the operation room (OR), that was 21 h after the hospital admission, with arterial Hb 5, pH 7.2 and lactate 20.9. Sternotomy was performed under ECMO support. Bradycardic broad QRS complexes and cardiac systolic ejection appeared after opening the pericardium and removing the massive pericardial clot (> 1000 ml). After full heparinisation, the peripheral ECMO cannulas were connected to the heart-lung machine and 6 units of packed red blood cells (PRBC) were administered immediately.

**Table 1:** Perioperative and operative patient data

	24 h pre-OP	1 h pre-OP	Skin Incision Pre-OP		Skin Closure Post-OP		6 h Post-OP	12 h Post-OP	24 h Post-OP	72 h post-OP
Patient	I	I	I	II	I	II	II	II	II	II
*Arterial BGA*	Air	P-Drain	ECMO	MV	ECMO	MV	I-CPAP	6 L O_2_	2 L O_2_	Air
pH	7.346	**7.097**	**7.021**	**7.184**	7.212	7.408	7.418	7.402	7.443	7.437
Hb,	11.9	11.2	**4.5**	14.1	7.0	9.4	9.6	9.9	8.8	8.5
Htc,	0.32	34.6	**14.5**	43.2	21.8	29.2	29.8	30.6	27.4	26.5
pO_2_, mmHg	59.7	98.4	401	188	300	194	125	88.7	95.8	97.2
pCO_2_, mmHg	35.9	22.7	32.2	24.6	47.1	40.2	40.2	45.6	37.8	39.3
Sat O_2_, %	92.3	93.2	98.9	98.8	99.2	99.1	99.1	96.3	96.9	97.3
Lactate, mmol/L	0.4	**13.9**	**20.9**	**9.6**	**18**	3.4	2.5	1.8	1.2	0.9
*Chemistry*										
Pro-BNP, pg/ml	697	1156		3256		2240				749
hs Troponin I	3602	2681		3006		5500	**11,000**	**16,000**	**13,000**	9000
CK-MB, U/L	**42**	25		< 24		**69**	**52**	47	37	15
CK, U/L	305	273		175		433	735	902	799	58
LDH,	501	377		**1454**		**2064**	**2359**	**1945**	**1094**	318
Potassium	4.3	4.6	**7.2**	5.1	**6.7**	**5.7**	5.0	4.5	4.1	4.0
ALT, U/L	62	46		**1256**		**1329**	**1136**	**774**	209	122
Bilirubin,	0.43	0.95		0.94		0.59	0.63	0.59	0.46	0.41
Creatinine, mg/dl	0.85	0.98		1.31		1.20	1.37	1.07	0.98	0.77
WBC, k/µl	11.2	13.2		15.4		20.9	18.2	15.8	11.4	9.3
CRP, mg/dl	174.8	217		49.9		33.0	132.6	222.6	156.9	108.6
Myoglobin,	49	88		**244**		**711**	368	269		

Legend

pre-, intra- and post-operative (OP) laboratory data of patient I (I) and patient II (II).

P-Drain, pericardial drainage; MV, mechanical ventilation; I-CPAP, invasive CPAP; 6 L O2, 6 L/min nasal oxygen supplementation;

ECMO, peripheral veno-arterial extracorporeal membrane oxygenator

Inspection of the unloaded heart on cardiopulmonary bypass (CPB) revealed a 2.5 cm long LVFWR within a 1 × 3.5 cm necrotic area in the region of distribution of the first obtuse branch of the circumflex artery (coronary segment 12) confirming with the previous angiography (Fig. 1). Coronary calcification was excluded by inspection and palpation. Epicardial echography showed significantly reduced global biventricular function with akinesia of the lateral wall of the left ventricle and ruled out valvular abnormalities.

Aortic cross-clamping and cardioplegic cardiac arrest was instituted by intermittent antegrade and retrograde administration of blood warm (Calafiore) cardioplegia. Surgical repair of the left ventricular tear was performed by linear closure with Prolene 4/0 as a horizontal mattress sutures with two supporting 4 cm long Teflon-felt strips. A second patch of Teflon was sealed on the suture line using surgical glue and fixed with an additional running Prolene 4/0 suture on the mattress suture. A hemostatic TachoSil (hemostatic fleece) was applied to widely cover the suture and the adjacent left ventricular tissue. Controlled aortic root reperfusion was instituted for 10 min. No electrical activity was assessed. Aortic declamping followed. Reperfusion was continued under epicardial DDD-pacing and lung-ventilation. Further transfusion and adequate ultrafiltration were performed (Table 2). Although trasesophageal echocardiography ruled out valvular dysfunction or regional myocardial dyskinesia, weaning from the CPB was not possible due to persistent biventricular cardiac failure. The ECG showed a bradycardic broad QRS ventricular rhythm hardly reacting to DDD-pacing. After 60 min of reperfusion, CPB circulation was switched back to veno-arterial ECMO support. This maneuver was not well tolerated by the patient, and shortly thereafter cardiac arrest was assessed in echography. Although massive inotropic and chronotropic support with Dobutamin, Milrinon, Noradrenalin, Vasopressin and finally Adrenalin was applied, stable hemodynamics could not be achieved. Venous return decreased progressively and continuous volume substitution was necessary to maintain ECMO flow. Intraabdominal free fluid accumulation was not detected by sonography. No significant intrathoracic bleeding was observed. Lactate values remained high in spite of the extensive therapy (Table). Massive volume administration failed to improve circulation and the patient died 50 min later under maximal pharmacological support with ECMO-flow decreasing below 1 L/min, blood pressures below 50 mmHg and lack of electrical response to maximal epicardial pacing.

**Table 2:** Intraoperative patient data

	Surgery			Surgery	
Patient	I	II	Patient	I	II
*Transfusion*			*Operative Data*		
PRBC (Unit)	**24**	-	OP-Time (min)	235	170
FFP (Unit)	**6**	3	CPB (min)	130	79
TC (Unit)	**2**	-	Cardiac Arrest (min)	55	36
PCC (IU)	3000	2000	Lowest temp. (°C )	**30**	**35**
Fibrinogen (g)	**8**	2	Filtration (ml)	2800	1200
Autologous (ml)	**1158**	450	Fluid Balance (ml)	2951	1929

Legend

Operative (OP) data of patient I (I) and patient II (II).

PRBC; packed red blood cells; FFP, fresh frozen plasma; TC, thrombocyte concentrate;

PCC, Prothrombin complex concentrate for emergency anticoagulation (Beriplex P/N, CSL Behring, Marburg, Germany); IU, international unit

### Patient II

A 76 year-old male patient without previous medical history and no known cardiovascular disease presented to the emergency department of a district hospital due to sudden syncope and progressive dyspnea. Upon admission the patient shows arterial hypotension (96/53 mmHg), tachycardia (88beats/min), jugular venous congestion, respiratory frequency of 18/min, peripheral O_2_ saturation 90% under mask oxygenation with 10 L 100% O_2_. He is somnolent, acidotic (pH 7.184) with elevated lactate (arterial 8.4 mmol/L, venous 9.6 mmol/L).

**Fig. 3:**
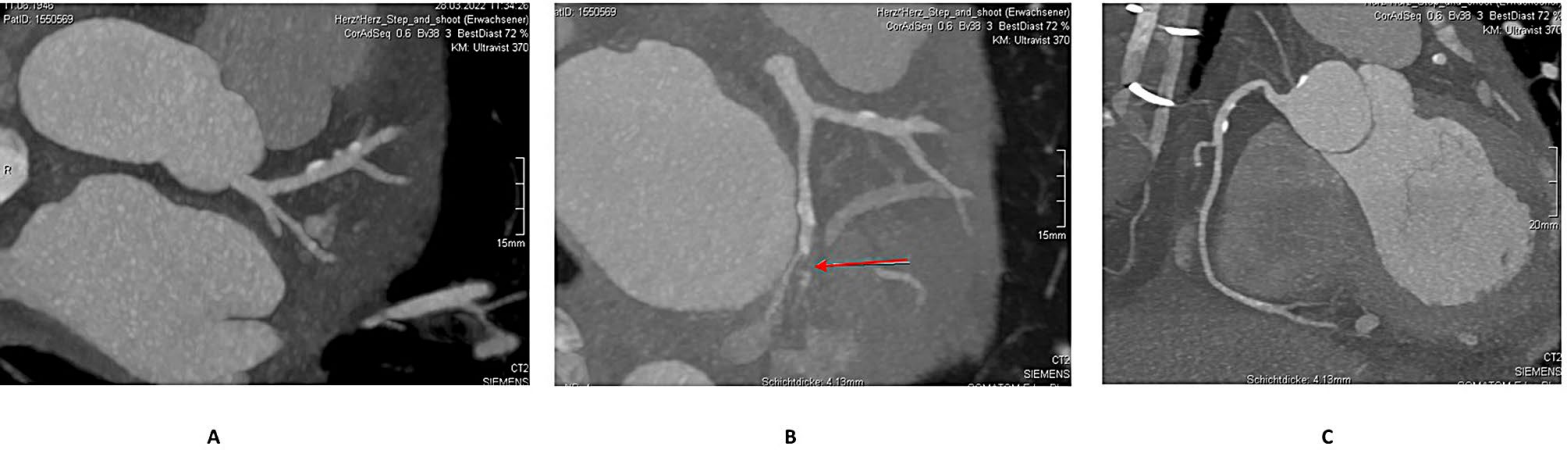
Preoperative screening for coronary disease in patient II Coronary thoracic CT-angiogram reconstruction of patient II reveals an intact main left coronary artery (Fig. 3A), suspicion (red arrow) of an occluded first marginal branch (Fig. 3B), and an intact right dominat coronary artery (Fig. 4C)

Transthoracic point-of-care echocardiogram showed relevant circumferential pericardial effusion (> 2 cm) with compression of the right atrium and ventricle. The patient was emergently transferred to our university department with the suspicion of Type A acute aortic dissection under increasing continuous catecholamine administration (achieving 40 µg/min noradrenaline). The ECG showed no signs of acute myocardial infarction. The emergent CT-Angiography (Fig. 2B) revealed normal ascending and descending thoracic aorta with no signs of dissection, a hemodynamically relevant (> 700 ml) pericardial effusion (red arrow) and a hypocontrasted myocardial area (3 × 2 × 2 cm) on the lateral left ventricular wall (red circle). Three-dimensional reconstruction of the coronary CT-angiography revealed normal coronary ostia with minimal calcification of the main branches (Fig. 3A, B and C). High-sensitivity cardiac troponin I was 3006.2ng/L and CK-MB < 24U/L.

The patient was increasingly unstable during the examinations, with progression of hypotonia and tachycardia in spite of the higher catecholamine substitution (80 µg/min Noradrenaline). Under the diagnosis of critical pericardial tamponade emergency operation was indicated and the patient was immediately transferred to the OR without previous coronary angiogram, and arrived in the OR 2 h after his presentation to the district hospital with laboratory revealing Hb 14, pH 7.2 and lactate 9.8. The patient remained awake and breathing independently with oxygen mask.

Insertion of the central venous and peripheral arterial lines, disinfection, draping and preparing the operating table for CPB took place before initiation of anesthesia. Asystole occurred during relaxation and endotracheal intubation. Sternotomy was performed under intermittent mechanic resuscitation in less than 2 min from the time of endotracheal intubation. Spontaneous circulation recovered immediately after pericardiotomy and removal of clot (> 800 ml). Sinus rhythm and elevated blood pressure occurred after 1000 mL crystalloid volume administration. Inotropic support was paused. Inspection of the heart revealed a 2.0 cm long LVFWR within a 1 × 3.5 cm necrotic area in the region of distribution of the first obtuse branch of the circumflex artery (coronary segment 12) and conforming with the hypoperfused territory detected on the CT scan (Fig. 2B). Inspection and palpation revealed a right dominant coronary system with no signs of coronary calcification. Transesophageal echocardiography showed good biventricular function, normal coronary ostia, normal ventricular wall motion and ruled out valvular pathology. Cannulation of the ascending aorta and double stage right atrial cannulation were performed and CPB instituted.

Aortic cross-clamping and cardioplegic cardiac arrest was instituted by intermittent antegrade and retrograde administration of blood warm (Calafiore) cardioplegia. Surgical repair of the LVFWR was performed by linear closure with a Prolene 4/0 horizontal mattress sutures with two supporting 3.5 cm long Teflon-felt strips. Surgical glue was applied along the suture line and a TachoSil fleece was applied to cover the suture and the additional left ventricular tissue.

Controlled aortic root reperfusion was instituted for 5 min and a normo-frequent sinus rhythm was assessed. Aortic declamping and transition from mechanical pump-assisted circulation to spontaneous heart activity was easily achieved with sufficient blood flow to maintain systemic circulation, under minimal catecholamine support. Weaning from CPB was performed under echographic surveillance. Good biventricular function without wall dyskinesia was assessed. Valve function was normal. Three units of FFP and 2 gr Fibrinogen were administered after ending CPB (Table 2). Lactate fell below 4 mmol/L (Table 1). Decannulation, hemostasis, sternal and wound closure were performed. The patient remained stable and was transferred to the ICU under minimal catecholamine support. Extubation followed 6 h postoperatively. Neurological examination was flawless. Oral nutrition and mobilization were initiated. The patient was transferred to the ward on the second postoperative day and to the rehabilitation on the 7th postoperative day. Coronary CT-scan performed before hospital discharge showed minimal and localized coronary calcifications without relevant stenosis. Full clinical recovery was achieved. Twelve months postoperatively, the patient is living at home symptom-free, in sinus rhythm, with normal biventricular pump function on aspirin, statin and minimal beta-blocker therapy.

## Discussion

The treatment of LVFWR caused by myocardial ischemia on the vascular territory of the first marginal branch of the circumflex artery in the absence of diffuse coronary atherosclerosis and without the possibility of coronary revascularization remains challenging. Recent publications reveal that less-invasive treatment increases the risks of left ventricular pseudoaneurysm formation [8, 9] and the attempts to reduce surgical invasiveness by means of percutaneous cardiocirculatory support and drainage were unsuccessful [10].

In consent, the remarkably different presentation of our clinical cases leaded to the use of different therapeutic approaches at the time of hospital admission.

Whereas coronary angiography excluded treatable atherosclerotic coronary disease and confirmed the occlusion of the first marginal branch without option of revascularization in patient I (Fig. 1), the CT scan raised the suspicion of a similar coronary branch occlusion in patient II (Fig. 3). Although contrast ventriculography (Fig. 1C) may have helped establishing the definitive diagnosis in patient I and could along with the coronary angiography facilitate surgical planning, this invasive procedure might be precarious by precipitating massive pericardial hemorrhage and tamponade. Cardiac-MRI [7] providing essential information on the characteristics of the ischemic lesion may help planning surgical intervention; nonetheless, extensive cardiac examinations are legitimate only in hemodynamically stable patients. Whether the information gained through additional imaging would substantially affect the choice of surgical repair and influence outcome of LVFWR remains rather uncertain. Sutureless repair of small LVFWR may be life-saving, however it can result in early development of a pseudoaneurym [8, 9] with all its local and systemic risks.

Whereas patient II required immediate surgery due to critical cardiac tamponade (Fig. 2B) with aggravating circulatory instability, patient I showed a localized coronary occlusion without possibility of revascularization, minimal pericardial effusion and decreasing cardiac enzyme levels. Conceivably, when the suspicion of pseudoaneurysm occurs without massive pericardial effusion, it is difficult to decide upon timing and the immediate necessity of surgical correction, although the onset of rupture remains unpredictable. Pericardial centesis may relieve cardiac tamponade and improve circulation in the critical situation of LVFWR; however, this tool is often questionable because non-drainable clots take up much of the pericardial space. A recent publication of Kato et al. [10] describes that even attempting to achieve preoperative circulatory stability in a patient with LVFWR by connecting the pericardial drainage to the peripheral VA-ECMO remained unsuccessful. Similarly, pericardial centesis was not considered as best option to relief the worsening hemodynamical instability in our patient II. Because pericardial tamponade occurred abruptly in patient I, it was believed to be caused by a sudden and relevant fresh amount of intra-pericardial blood. However, immediate pericardial centesis remained hemodynamically ineffective and did not improve resuscitation, suggesting that a significant amount of clot was not removable. Nonetheless, in the presence of refractory cardiac arrest, ECMO provided the necessary bridging towards the chance to perform definitive surgical treatment in patient (I) Restoration of a satisfactory hemodynamic state on the way to surgery is indeed crucial. Thereby, patient I arrived in OR on ECMO, in profound and prolonged cardiogenic and hemorrhagic shock, requiring transfusion, massive inotropic support and buffer administration, while patient II necessitated a short period of mechanical resuscitation at the time of intubation recovering fully after opening the chest and release of the pericardial tamponade. Patient I had a deeply reduced global cardiac pump function after opening the pericardium, whereas at a similar time point biventricular pump function recovered entirely in patient (II) More importantly, although both patients had significantly elevated serum lactate levels (Table 1) indicating severe shock at the time of sternotomy, electromechanical function remained deeply impaired after the removal of the pericardial tamponade in patient I, whereas electrical conduction normalized entirely in patient II. Alike the findings of Kato et al. [10], pericardial drainage and ECMO support seemed less effective than expected and prolonged somehow the period of circulatory instability of our patient I. Although percutaneous circulatory support with ECMO or Impella may shorten the duration of circulatory shock [2, 10, 15] and facilitate a time-interval for diagnostic and preparation to surgery in hemodynamically instable LVFWR, the value of extensive preoperative diagnostic should be waged against and the outcome of rapid surgical intervention [11].

Whereas right ventricular free wall rupture caused by acute right coronary artery disease may resolve with moderate less-invasive treatment [3]. Conservative treatment of LVFWR [4] has as direct consequence the late development of pseudoaneurysm, impairing left ventricular pump function [5–7, 12] more often than the right ventricular rupture [13]. Moreover, studies show that the surgical repair of LVFWR by means of glue application and local compression off-pump [14], might not be as effective on long term as the direct suture on CPB or the resection of the necrotic area with patch replacement on cardiac arrest [15].

In our both LVFWR cases, surgical repair involved closure of the tear and exclusion of the small surrounding necrotic myocardial territory susceptive to rupture. Cardiopulmonary bypass with cardiac arrest enabled precise positioning of the sutures and technically successful closure of the myocardial tear with minimal loss of viable myocardium and a reduce risk of coronary damage. In addition, cardiac arrest makes possible grafting of any newly and intraoperatively identified and surgically treatable left lateral wall coronary artery branch distal from LVFWR.

In agreement with the previous publications of Matteucci et al. [2, 15], our present experience asserts that treating pericardial tamponade by prompt sternotomy and digital control of the rupture seem to terminate cardiogenic shock, reduce the duration of circulatory collapse and diminishe the risk of exsanguination. Our findings highlight that an emergency surgical procedure can sufficiently be guided by transesophageal echocardiography. The current guidelines [6] acknowledge the effectiveness of intraoperative transesophageal echocardiography in identifying wall motion disturbances, dysfunction of the cardiac valves and relevant pathologies of the coronary origins. Direct intraoperative assessment of the necrotic area and identification of coronary calcification by inspection and palpation may be rigorously performed on CPB. The quality of the surgical repair is dependent of the unloading of the heart and cardiac arrest. Blood cardioplegia reduces the degree of hemodilution and the use of retrograde cardioplegic delivery as adjunct to antegrade delivery protects the myocardial areas distal from rupture as well as the myocardial areas eventually hypo-perfused due to yet unknown coronary artery stenosis. Newly appeared wall motion disturbances during reperfusion of the heart may warrant grafting of the involved coronary branches, and this may be easily accomplished on beating heart.

Altogether, the present cases indicate that adequate surgical reconstruction of a LVFWR and complication-free cardiac surgery can be safe also without extensive preoperative diagnostic.

Preoperative coronary angiography and MRI [2, 7] may improve planning the type and magnitude of surgery, however, one should keep in mind that the final decisions regarding the details of the most appropriate surgical repair are determined based on the intraoperative findings [15].

## Conclusions

Local ischemic ventricular rupture without treatable atherosclerotic coronary disease is not associated with poor cardiac pump function and therefore, surgical reconstruction under cardiac arrest is very likely to be successful.

Poor outcome after LVFWR repair may strongly be associated to circulatory shock resulting from hypovolemia, and to acute cardiac failure caused by intrapericardial bleeding and tamponade.

Early surgical repair leads to best outcome suggesting that treating LVFWR as a high emergency indifferent from symptoms may improve survival.

## Data Availability

The authors confirm that the included images and associated text, has been obtained from the patient recordings, in line with COPE guidance. The anonymized clinical details and/or clinical images are available for review.

## References

[1] Yip HK, Wu CJ, Chang HW, Wang CP, Cheng CI, Chua S (2003). Cardiac rupture complicating acute myocardial infarction in the direct percutaneous coronary intervention reperfusion era. Chest.

[2] Matteucci M, Formica F, Kowalewski M, Massimi G, Ronco D, Beghi C (2021). Meta-analysis of surgical treatment for postinfarction left ventricular free-wall rupture. J Card Surg.

[3] Hasnie UA, Wagner C, Elrod JP, Chapman GD (2021). Right ventricular Free Wall Rupture after myocardial infarction. JACC Case Rep.

[4] Dewulf M, Cathenis K, Goossens D (2015). Conservative Treatment of Left Ventricular Free Wall Rupture. Acta Chir Belg.

[5] Roelandt JR, Sutherland GR, Yoshida K, Yoshikawa J (1988). Improved diagnosis and characterization of left ventricular pseudoaneurysm by Doppler color flow imaging. J Am Coll Cardiol.

[6] Lancellotti P, Price S, Edvardsen T, Cosyns B, Neskovic AN, Dulgheru R (2015). The use of echocardiography in acute cardiovascular care: recommendations of the European Association of Cardiovascular Imaging and the Acute Cardiovascular Care Association Eur. Heart J Cardiovasc Imaging.

[7] Del Monte A, Perazzolo Marra M, Cardaioli F, Gerosa G, Iliceto S, Cacciavillani L (2022). Two left ventricular pseudoaneurysms complicating a myocardial infarction: the impact of Cardiac magnetic resonance in the Acute setting. Can J Cardiol.

[8] Uchino M, Yoshikai M, Miho T, Koga K, Amamoto S (2023). Ventricular pseudoaneurysm after Sutureless Repair of Postinfarction Free Wall rupture of the left ventricle: report of a case. Kyobu Geka.

[9] Mukkannavar SB, Pai TJ, Ramesh NR, Radhika TK, Dhanasekaran KS (2023). Sutureless repair of left ventricular free wall rupture following acute myocardial infarction with cardiogenic shock. Indian J Thorac Cardiovasc Surg.

[10] Kato T, Miyagawa A, Hikone M, Yuri K, Sugiyama K (2023). Peripheral VA-ECMO and pericardial drainage connected to the ECMO circuit for cardiac tamponade from blowout rupture: a case report. BMC Cardiovasc Disord.

[11] Kim HK, Kim SS, Choi IY, Ki YJ, Park KH, Choi DH (2023). Left ventricular Free Wall Rupture immediately following successful coronary reperfusion complicating ST Elevation myocardial infarction. Chonnam Med J.

[12] Naseerullah FS, Pyle W, Addai T, Murthy A (2023). Left ventricular pseudoaneurysm without antecedent myocardial infarction. J Cardiol Cases.

[13] Sano Y, Kato T, Takama N, Hisanaga E, Matsumoto N, Amanai S (2022). Oozing-type rupture caused by right ventricular intramural hematoma after right ventricular infarction. J Cardiol Cases.

[14] Canovas SJ, Lim E, Dalmau MJ, Bueno M, Buendía J, Hornero F (2003). Midterm clinical and echocardiographic results with patch glue repair of left ventricular free wall rupture. Circulation.

[15] Matteucci M, Fina D, Jiritano F, Meani P, Blankesteijn WM, Raffa GM (2019). Treatment strategies for post-infarction left ventricular free-wall rupture. Eur Heart J Acute Cardiovasc Care.

